# The Regulation of Trypanosome Gene Expression by RNA-Binding Proteins

**DOI:** 10.1371/journal.ppat.1003680

**Published:** 2013-11-07

**Authors:** Christine Clayton

**Affiliations:** Zentrum für Molekulare Biologie der Universität Heidelberg (ZMBH), DKFZ-ZMBH Alliance, Heidelberg, Germany; University of Wisconsin Medical School, United States of America

## Trypanosomes Depend on Post-transcriptional Mechanisms to Regulate Gene Expression

The genome organization of trypanosomes, leishmanias, and related kinetoplastid organisms is highly unusual: Each RNA polymerase II promoter precedes multiple open reading frames, and the primary transcript is cut into individual mRNAs by 5′ *trans* splicing and 3′ polyadenylation [1]. Dedicated control of transcription at the level of individual mRNAs is therefore not possible. Nevertheless, mRNA levels are highly controlled: Although some gene sequences are represented by several hundred mRNAs, most mRNAs are present at just one or two copies per cell [2]. Constitutively high mRNA levels can be obtained by having many identical genes, but for most genes, more flexible control points are used: mRNA processing, export from the nucleus, translation, and mRNA decay.

Kinetoplastid mRNAs are probably bound by multiple proteins possessing varying degrees of sequence specificity. The poly(A) tail is masked by poly(A) binding proteins (PABP1 and 2). During translation the 5′ cap is bound by initiation factors [3], [4]; it is expected that, as in other eukaryotes, these interact with PABP, increasing translation efficiency and shielding the mRNA from degradation. Multiple other proteins could bind both translated and untranslated regions, with potential competition between proteins for particular binding sites. Interactions between RNA-binding proteins and proteins of the processing, translation, and degradation machineries will combine to determine RNA fate.

This review focuses on *Trypanosoma brucei*. *T. brucei* has two life-cycle stages that grow in culture in the laboratory: the bloodstream form (similar to that in mammalian blood) and the procyclic form (similar to that in the midgut of Tsetse flies). Differentiation of bloodstream forms to procyclics occurs via an intermediate, non-dividing stage, the stumpy form. In Tsetse, the procyclic form in the midgut differentiates to epimastigotes, then to mammalian-infective metacyclic forms in the salivary glands. Methods used for the investigation of RNA-binding proteins are listed in Table 1.

**Table 1:** Methods that can be used to study the functions of mRNA-binding proteins.

Question	Approach	Comments	Examples
A. Which protein to study?	1. In silico identification of proteins with predicted RNA-binding domains	Not all proteins with canonical RNA-binding motifs bind to RNA.	Most proteins studied
	2. Use a specific RNA as a ligand to purify proteins that bind to it	Can result in purification of relatively abundant proteins with low RNA-binding specificity.	ALBA1-4 DRBD3
	3. Use a gel shift assay with a chosen RNA to monitor purification of a binding protein		
	4. Cross-link protein to a reporter RNA in vivo, then use that RNA to pull out the RNA-protein complex [31], [32]	Technically challenging, few examples of successful application.	None so far
	5. Genome-wide screens	RNAi screens work only if the encoded protein is not essential.	NRG1
B. Which RNAs does the protein bind?	1. RNA-IP: UV-Cross-link protein to RNA *in vivo*. Purify the protein with attached RNA. Identify RNAs using RNAseq or microarray.	Proteins may associate with RNA after cell lysis. Protein over-expression can lead to less specific binding.	PUF9, ZC3H11, ZFP3
	2. Variant of B-1: limited RNase digestion prior to purification allows identification of the binding site	Current gold standard, technically challenging.	DRBD3, RBP42
	3. Pull down protein then test binding of selected mRNAs by RT-PCR or Northern blot	Biased by choice of tested RNA sequences. Best if sequences are chosen using B-1 or B-2.	Many
	4. Test purified protein in gel-shift assays with various RNA sequences; use competition assays to determine specificity	Recombinant protein may not fold properly; risk of bias as for B-3.	Many
C. Where is the protein in the cell?	1. Express epitope-tagged protein, examine by immunofluorescence or cell fractionation	Tagging or over-expression can cause mis-localization.	Many
	2. Use specific antibody for immunofluorescence or cell fractionation	Cell fractionation is more sensitive than immunofluorescence.	Many
D. How much protein is present, relative to potential RNA targets?	1. Measure amount by Western blotting with antibody and recombinant protein standard	Check that any tag on the recombinant protein does not influence antibody detection.	ALBA1-4, UBP1,2.
	2. Tag one allele *in situ*, measure signal relative to another cell line in which the amount of tagged protein is known	Tag may affect protein stability.	ZC3H11
E. Is the protein associated with RNA during translation?	Sucrose density gradient fractionation of polysomes followed by Western blotting	Proteins may dissociate during centrifugation because of high dilution.	Many
F. Which mRNAs are influenced by the protein?	1. RNAi followed by transcriptome analysis (RNASeq or, previously microarray)	Growth inhibition after RNAi can have secondary effects on the transcriptome.	PUF9, ZC3H11, DRBD3, ZC3H20, HnRNPF/H, ALBA
	2. Inducibly (over)-express the protein followed by transcriptome analysis	Over-expressed protein may bind non-specifically, effects may be secondary, tagging can alter function.	ZFP3, RBP10, UBP2
	3. RNAi or over-express, Northern or real-time PCR measurement of individual mRNAs	Useful for confirmation of E-1, E-2. Biased if there is no transcriptome analysis.	Many
	4. RNAi followed by ribosome profiling [28]	Current gold standard to measure role of a protein in translation.	None
G. How does the protein affect overall phenotype?	1. RNAi, measure growth, cell morphology, cell cycle stages, ability to differentiate	Changes in cell-cycle pattern often non-specific.	All
	2. Knock out both alleles	Definitive although other proteins may take over function.	None so far
H. Does the protein affect mRNA stability?	Use cells with RNAi, inhibit transcription, measure selected mRNAs by quantitative RT-PCR or Northern blotting	Useful if mRNAs selected using E-1.	Many
I. Does the protein act via specific sequences in an mRNA?	Express reporter mRNA with and without test sequence, measure effects of RNAi on reporter expression	Distinguishes between effects on translation and transcript level. Regulatory sequence context often important.	Many
J. Does the protein interact with others?	1. Purify the protein (usually by a tandem affinity purification approach), identify the associated proteins by mass spectrometry	Only stable associations detected. Affinity tag or over-expression may affect protein function.	DRBD3, ALBA, RBP10, NRG1, PUF7, PUF10, BOP1
	2. Screen for interactions using the yeast two-hybrid system	Detects transient interactions but prone to false-positives.	None so far
	3. Examine individual interactions by co-immunoprecipitation	Can confirm interactions from J-1 or J-2 and check if they are RNA-dependent.	ALBA, NRG1, PUF7, PUF10, BOP1
K. Does the protein actively stabilize or destabilize mRNA?	Express protein fused to a specific RNA-binding domain (e.g. lambda N or MS2 peptide). Co-express a reporter RNA with the peptide binding site.	Protein fusion with the RNA-binding peptide, or binding via the peptide rather than via the normal RNA-binding domain, which may affect function.	ZC3H12, RBP10, ZC3H11

Examples of trypanosome proteins in the right-hand column are not exhaustive lists: For details of the results and references, see the text. Where the author is unaware of documented use for kinetoplastids, a reference for use in other organisms is provided.

## The Kinetics and Enzymes of mRNA Decay

Most bloodstream-form *T. brucei* mRNAs have half-lives of five to 20 minutes, but some higher abundance mRNAs have half-lives exceeding two hours; results for procyclics are quite similar ([2] and our unpublished results). The first step in degradation of most mRNAs is removal of the poly(A) tail by the CAF1/NOT complex [2], [5]. This will result in loss of PABP, exposing the mRNA to further degradation—removal of the cap, then degradation by the 5′-exoribonuclease XRNA [2] and/or from the 3′-end by the exosome complex. Depletion of XRNA by RNAi inhibited degradation of some unstable mRNAs; in contrast, a decrease in exosome abundance caused slight delays in degradation of some mRNAs with intermediate half-lives (20 to 60 minutes) [2], [5]. Although most mRNAs show simple exponential decay, others show biphasic kinetics, suggesting involvement of two different rate-limiting processes (our unpublished results).

An RNA-binding protein that interacts with XRNA could enhance 5′-3′ degradation, while one that recruits the CAF1/NOT complex should accelerate deadenylation. Other proteins could stabilize RNAs or enhance translation. They could do this via interactions with translation factors, or simply by preventing the binding of a protein that promotes degradation. This review focuses on selected RNA-binding proteins that influence mRNA levels or translation.

## Alba and Pumilio Domain Proteins

Four small proteins with an Alba domain, ALBA1, 2, 3, and 4, form homo- and heterodimers. ALBA2 and ALBA3 are associated with polysomes, and their depletion affects translation of a reporter in a sequence-specific fashion; ALBA3 also interacts with a translation initiation factor. Each ALBA protein is present at 10,000–20,000 molecules per procyclic trypanosome [6]. For comparison: Each procyclic trypanosome has about 40,000 mRNAs, with half as many in bloodstream forms [7]. The lower the protein-to-mRNA ratio, the higher the probability of sequence-specific interactions and function.

Of the ten *T. brucei* pumilio-domain proteins, PUF9 is the only one with a clearly defined role in mRNA degradation: It binds to, and is required for stability of, a small number of mRNAs that increase in the late G1 phase of the cell cycle [8]. A putative recognition motif, UUGUAC, was identified and shown to be required for PUF9-mediated regulation.

PUF7 and PUF10 are in the nucleolus and required for rRNA maturation, as are two other nucleolar proteins, BOP1 and NRG1 [9]. These four proteins show several mutual interactions. Intriguingly, depletion of any of them results in an increase in the level of the mRNA that encodes the GPEET procyclin surface protein at times when it is normally suppressed. This is especially interesting because the *GPEET* genes are transcribed by RNA polymerase I, in the nucleolus. NRG1 is also associated, either directly or indirectly, with the *GPEET* mRNA [9].

## RRM Domain Proteins

*T. brucei* has about 70 different proteins with at least one RRM (RNA Recognition Motif) [10]; of these, about half are essential for normal growth in at least one life-cycle stage [11]. The two poly(A) binding proteins, PABP1 and PABP2, are both associated with polysomes, but may nevertheless have different functions [12]. UBP1 and UBP2, which are similar proteins with a single RRM, are present in a 100-fold excess over mRNA [13], so are unlikely to have sequence-specific functions in vivo, although results from *Trypanosoma cruzi* indicate that they do show preference for some sequences over others [14]. UBP1 and UBP2 are essential, but a microarray analysis detected little effect on the transcriptome when *UBP1* and *UBP2* were targeted by RNAi. Over-expression caused transcriptome changes [13], but all results from over-expression must be viewed with extreme caution due to the likelihood of artifacts (Table 1, E2).

RBP42 has a single RRM and is partially associated with polysomes [15]. Its RNA target sites have been mapped accurately (Table 1, B2) [15]. Two thousand different mRNAs showed some binding; interestingly, the 188 that showed most enrichment in the RBP42-associated fraction included many that are involved in energy metabolism. RBP42 bound preferentially to coding regions rather than to untranslated regions [15]; how this is consistent with translation is not yet known. RBP42 is essential in all stages tested, but the effect of depletion on the transcriptome has not yet been described.

DRBD3 (also called PTB1), which has two RRMs, has roles in both splicing and mRNA stability. RNA interference caused moderate (1.5–3×) decreases in 21 different mRNAs, with enrichment for membrane proteins [16]. For a few targets, the decrease in mRNA was shown to be due to destabilization [16], [17], mediated via the 3′ untranslated region of the mRNA (3′-UTR) [16]; the latter observation correlates with the preference of DRBD3 for binding to 3′-UTR sequences [15]. Oddly for a stabilizing protein, DRBD3 was not detected in polysomes, although dissociation during gradient centrifugation has not been ruled out. Affinity purification revealed that DRBD3 interacts with two splicing factors, which correlates with its second likely function in splicing: DRBD3 is in both nucleus and cytoplasm, and RNAi targeting *DRBD3* caused clear increases in the levels of some mRNA precursors [17].

Two RRM-domain proteins have been implicated in life-cycle-stage-specific gene expression control. The *RBP6* mRNA is most abundant in epimastigotes. Remarkably, ectopic expression of RBP6 in procyclic forms promotes differentiation not only to epimastigotes, but also to the mammalian-infective metacyclic form [18]. RBP6 target mRNAs are not yet known. Meanwhile, the presence of RBP10 correlates with a bloodstream-form expression pattern, especially for genes of energy metabolism [19]. RBP10 RNAi in bloodstream forms decreases their expression, while artificial expression in procyclics increases the mRNA levels. The obvious interpretation is that RBP10 stabilizes energy metabolism mRNAs, but three other observations contradict this idea. Even using a sensitive assay, no binding of mRNA by RBP10 was detected, and it was not associated with polysomes. Moreover, artificial attachment of RBP10 to mRNAs inhibited their translation [19].

Finally, another RRM domain protein, HnRNPF/H, is able to influence both mRNA stability and splicing, with binding sites in 5′- or 3′-UTRs [20]. This provides a potential link between nuclear processing events and cytoplasmic mRNA regulation.

## Zinc-Finger Domain Proteins

There are about 40 *T. brucei* proteins with C-X8-C-X5-C-X3-H zinc finger domains. ZFP1 is procyclic-specific, while ZFP2 is expressed in bloodstream forms as well; both are required for differentiation of the bloodstream form to the procyclic form [21], [22]. A global analysis of mRNAs associated with ZFP3 in procyclics [23] revealed a bias towards mRNAs that are increased in the stumpy form. ZFP3 also binds specifically to some mRNAs encoding procyclic surface coat proteins, with over-expression promoting translation [24] and RNAi decreasing target mRNA levels.

ZC3H20 is required for growth of procyclic forms [25]. After depletion by RNAi, 12 RNAs were decreased; the two tested were, as expected, destabilized and the effect could be assigned to their 3′-UTRs. Binding of ZC3H20 to two target mRNAs was also demonstrated.

ZC3H11 is essential in bloodstream forms. It is not required for procyclic growth at 27°C, but is required at 37°C, and for the parasites to survive a transient 41°C heat shock [26]. Correspondingly, ZC3H11 binds to, and stabilizes, mRNAs that encode the complete set of chaperones needed for protein refolding after heat denaturation. The N-terminal zinc finger binds specifically to (UAU) repeats in the mRNA 3′-UTRs, while the C-terminal domain has the stabilizing activity [26]. At the normal growth temperature in both forms, ZC3H11 is almost undetectable, but the amount of protein increases dramatically during heat shock. ZC3H11 is the only RNA-binding protein for which a mechanism is known. It interacts with two other proteins, MKT1 and PBP1; PBP1 in turn interacts with PABP (unpublished results). Recruitment of PABP to the 3′-UTR is already known to greatly increase mRNA stability [27]. Thus ZC3H11 could act as a platform to recruit PABP to the 3′-UTR.

## Conclusions and Perspectives

Our knowledge of the functions of mRNA-binding proteins in *T. brucei* is still very fragmented and almost entirely restricted to those with predicted RNA-binding domains. Only a minority of such proteins has been assessed at a transcriptome-wide level for roles in determining mRNA abundance, and global analysis of translation [28] has not yet been applied. Moreover, by restricting investigation to proteins with known RNA binding domains, many important regulators are likely to be missed, since surveys of mRNA-bound proteins in other organisms have revealed that many lack such domains [29], [30].

By comparison with yeast and mammalian cells [29], [30], we can expect 100–200 different proteins to be associated with trypanosome mRNA. Different sets of proteins, with varying degrees of specificity, will be associated with different sequences, competing and cooperating with each other and changing over the mRNA lifetime. In the future, it will be essential to move from studies of individual proteins towards understanding the complex networks of interactions that determine the fates of individual mRNAs.
